# Structural and developmental expression of *Ss-riok-*2, an RIO protein kinase encoding gene of *Strongyloides stercoralis*

**DOI:** 10.1038/s41598-017-07991-2

**Published:** 2017-08-18

**Authors:** Wei-Qiang Lei, James B. Lok, Wang Yuan, Yue-Zhou Zhang, Jonathan D. Stoltzfus, Robin B. Gasser, Si-Yuan He, Huan Zhou, Rui Zhou, Jun-Long Zhao, Min Hu

**Affiliations:** 1 State Key Laboratory of Agricultural Microbiology, Key Laboratory of Development of Veterinary Diagnostic Products, Ministry of Agriculture, College of Veterinary Medicine, Huazhong Agricultural University, 1 Shizishan Street, Wuhan, 430070 China; 2 Department of Pathobiology, School of Veterinary Medicine, University of Pennsylvania, 3800 Spruce Street, Philadelphia, PA 19104 USA; 3 Faculty of Veterinary and Agricultural Sciences, The University of Melbourne, Corner of Flemington Road and Park Drive, Parkville, Victoria 3010 Australia; 4 Department of Biology, Millersville University, Millersville, Pennsylvania USA

## Abstract

RIO kinases are essential atypical protein kinases in diverse prokaryotic and eukaryotic organisms, playing significant roles in yeast and humans. However, little is known about their functions in parasitic nematodes. In the present study, we have isolated and characterized the full-length cDNA, gDNA and a putative promoter of a RIOK-2 protein kinase (*Ss*-RIOK-2) encoding gene (*Ss-riok-*2) from *Strongyloides stercoralis*, a medically important parasitic nematode (Order Rhabditida). A three-dimensional structure (3D) model of *Ss-*RIOK-2 was generated using the *Chaetomium thermophilum* RIOK-2 protein kinase (*Ct-*RIOK-2) crystal structure 4GYG as a template. A docking study revealed some critical sites for ATP binding and metal binding. The putative promoter of *Ss-riok-2* contains a number of conserved elements. RNAseq analysis revealed the highest levels of the *Ss-riok-2* transcript in free-living females and parasitic females. To identify anatomical patterns of *Ss-riok-2* expression in *S. stercoralis*, we observed expression patterns of a transgene construct encoding green fluorescent protein under the *Ss-riok-2* promoter in post free-living *S. stercoralis*. Expression driven by this promoter predominated in intestinal cells. This study demonstrates significant advancement in molecular and cellular biological study of *S. stercoralis* and of parasitic nematodes generally, and provides a foundation for further functional genomic studies.

## Introduction

The atypical serine protein kinase RIOK-2, one of four members of the RIOK family (RIOK-1, RIOK-2, RIOK-3 and RIOK-B), named for the right open reading frame 1 domain in the prototype member of this family, RIOK-1 from yeast^1^. RIOK-2 is essential for its regulatory functions in ribosome biogenesis and other cell cycle events^2–6^. Among the four RIOK protein kinases, RIOK-2 has been observed in eubacteria, archaea and many eukaryotes including humans, yeast and *Plasmodium* spp.^2, 6–9^. Crystal structures of *Afu*-RIOK-2 from *Archaeoglobus fuldigus* (Archaea) and *Cth*-RIOK-2 from *Chaetomium thermophilum* (a thermophilic fungus) reveal that RIOK-2 consists of a conserved N-terminal winged helix (wHTH) domain and a RIO domain structurally homologous to atypical protein kinases. The N-terminal domain of RIOK-2 is similar to a nucleic acid-binding motif common to many DNA-binding proteins that contain a winged helix fold, which mediates protein-protein interactions and protein-RNA interactions^10–12^. Another structural feature found exclusively in the C-terminal domains of eukaryotic RIOK-2s is a conserved extension sequence^4^.

In yeast, RIOK-2 occurs in both the nucleus and cytoplasm^4^. In human cells, RIOK-2 (hRIOK-2) localizes to the cytoplasm of cells at steady state but rapidly accumulates in the nucleus when the exportin CRM1 (chromosome region maintenance 1) protein is inhibited by leptomycin B^6^. There is evidence that RIOK-2 is released to the cytoplasm after binding CRM1 at a functional leucine-rich nulear export signal in hRIOK-2′s C-terminal domain via RanGTP^13, 14^. Although the function of RIOK-2 in nuclear steps of biogenesis is presently unclear, RIOK-2 also has an important function in ribosome biogenesis after nuclear export. In both yeast and human cells, RIOK-2 is a non-ribosomal factor necessary for processing the 18 S precursor ribosomal RNA (pre-rRNA) and for cytoplasmic maturation of the 40 S ribosomal subunit. RIOK-2 is also a trans-acting factor involved in late steps of 40 S biogenesis^2, 4, 5^. In human cells, RIOK-2 is required for the processing of 18 S pre-rRNA, and for the release of ENP-1 (essential nuclear protein 1, a 40S-associated trans-acting factor) from cytopla smic 40 S precursors, and for the recycling of DIM-2 (defective entry into mitosis 2), LTV-1 (low temperature viability Protein 1) and NOB-1 (nin one binding protein 1), which are additional 40S-associated trans-acting factors^6^.

In *Chaetomium thermophilum*, kinase activity of RIOK-2 is identified as the enzyme is released from the late pre-40S particle via its catalytic activity and thus influences the progression and recycling of other late pre-40S processing factors. Moreover, loss of kinase activity results in significant growth defects and cold sensitivity in *C. thermophilum* ^15^. In human HeLa cells, phosphorylation of RIOK-2 by PLK-1 (Polo-like Kinase 1, encoding an essential gene in the maintenance of the mitotic progression) at Ser-335, Ser-380 and Ser-548 regulated the programmed transition of the cell cycle from metaphase to anaphase. RIOK-2 over-expression causes prolonged cessation of mitosis whereas knockdown of RIOK-2 can accelerate mitotic progression^16^. However, in glioblastoma cells, over-expression of RIOK-2 can also promote tumorigenesis by acting upstream of Akt signaling to stimulate Tor-complex-2 (TORC2) signaling^17^. This suggests that RIOK-2 may have crucial but different functions in complex signaling events in diverse cells to regulate the entire cell-cycle.

In *Plasmodium* spp, the RIOK-2 kinase is also conserved among the 65 protein kinases in this parasite^8, 18^. The peptide binding sites and protein kinase inhibitor binding domain of *Plasmodium falciparum* RIOK-2 kinase highlights this molecule as a potential druggable target^19, 20^. The genome of the free-living nematode *C. elegans* encodes a RIOK-2, and silencing of *Ce-riok-2* by RNAi leads to embryonic lethality and sterility (http://www.wormbase.org/species/c_elegans/gene/WBGene00013688). This suggests that RIOK-2 is essential for the development and reproduction of this worm. The necessity of RIOK-2 for larval development in *C. elegans* opens the question of its potential as an essential regulator of development in parasitic nematodes. Consequently, we are characterizing a *riok-2* homolog in the intestinal parasitic nematode *Strongyloides stercoralis. S. stercoralis*, a parasite of dogs and humans, causes chronic debilitation in uncomplicated infections, and, by virtue of its ability to undertake cycles of autoinfection, can precipitate a potentially fatal disseminated hyperinfection in patients co-infected with HTLV-1 or undergoing corticosteroid therapy^21^. Human strongyloidiasis is grouped among the “neglected tropical diseases”^22^. *Strongyloides* spp. also offer significant advantages as subjects for functional genomic study, having published genome sequences^23^ and being amenable to transgenesis using techniques adapted from *C. elegans* biology^24^.

In the present paper, we report the discovery of *Ss-riok-2* in *S. stercoralis*. As first steps in characterizing the structure and function of this gene, we have isolated and sequenced cDNA corresponding to *Ss-riok-2* homolog, the gDNA and the putative promoter for this gene. We also explored the temporal and spatial patterns of *Ss-riok-2* expression. The primary objective of this study was to uncover clues to the function of *Ss-riok-2* with an eye to deducing its role in the biology of *Strongyloides* spp.

## Results

### Characterisation of *Ss-riok-2* cDNA and the evolutionary relationship of *Ss-*RIOK-2 to its homologues from other species

The full-length coding region of *Ss-riok-2* is 1572 bp in length, encoding 523 aa. The coding sequence of *Ss-riok-2* has an A + T content of 69.21%, and comprises two exons. Comparison of *Ss-*RIOK-2 with its homologues showed that *Ss-*RIOK-2 has significant similarities to RIOK-2s from a range of organisms, including three nematodes (*L. loa; C. elegans; H. contortus*) and six other organisms (*A. florea; H. sapiens; D. rerio; C. familiaris; A. fulgidus*; *S. cerevisiae*). The highest amino acid similarity recorded was to the *Hc-*RIOK-2 from *H. contortus*. Pairwise comparisons of amino acid sequence between *Ss-*RIOK-2 and selected sequences revealed sequence identities ranging from 36.99% to 45.94%.

**Figure 1:**
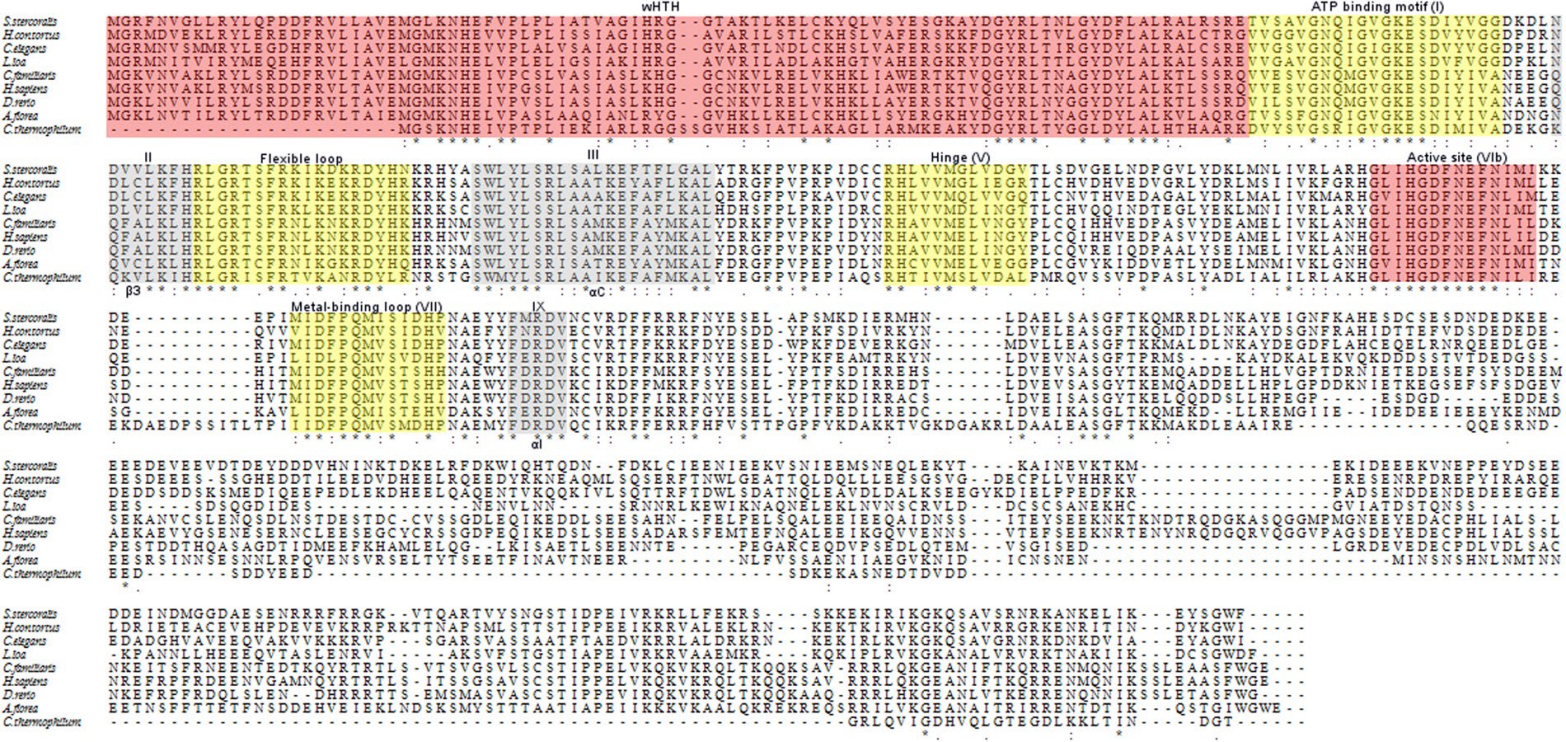
Multiple sequence alignment among predicted RIOK-2 proteins from eight organisms (*Strongyloides stercorali*s, *Loa loa*, *Haemonchus* *contortus*, *Caenorhabditis elegans*, *Apis florea*, *Danio rerio*, *Canis familiaris*, *Homo sapiens*, *Chaetomium thermophilum*). The N-terminal domain contains the conserved wHTH do main (red). The RIO kinase domain contains the ATP-binding motif (yellow), the flexible loop (yellow), the hinge region (yellow), and the catalytic (red) and metal-binding loops (yellow) as determined from the structure of the Archaeoglobus fulgidus RIOK-2 protein. The predicted subdomains I-IX are marked above the alignment. Asterisks indicate identical residues. Dashes indicate gaps in the sequence, included for alignment purposes.

The alignment of the amino acid sequences of *Ss-*RIOK-2 with selected RIOK-2 kinases (Fig. 1) showed conserved regions in the ATP-binding motif (sub-domain I), the hinge region (subdomain V), the active site (subdomain VIb), the metal binding loop (subdomain VII), the flexible loop inserted between the third β strand and the α helix C, and other subdomains, including II, III, IX. This level of conservation suggests that *Ss-*RIOK-2 is functionally similar to other RIOK-2 kinases. In addition, *Ss-*RIOK-2 contains the specific phosphate-binding loops of subfamily RIOK-2 domains (with the sequence GxGKES) in the ATP binding motif and a highly conserved N-terminal winged-helix (wHTH) domain, the signature characteristic of the RIOK-2 kinase family. The alignment also showed that the amino acid sequences in regions external to these subdomains are more divergent (Fig. 1) than the sequences in the subdomains.

**Figure 2:**
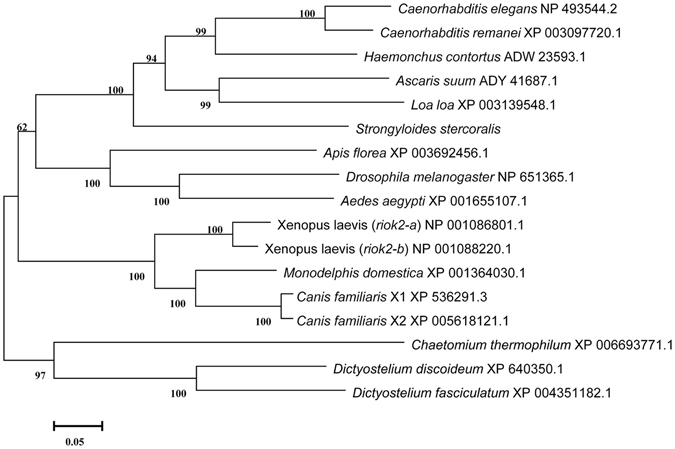
The neighbour-joining tree of RIOK-2 amino acid sequences from a range of organisms. These species are *Ascaris suum* (ADY41687.1), *Caenorhabditis elegans* (NP_493544.2), *Caenorhabditis remanei* (XP_003097720.1), *Haemonchus contortus* (ADW23593.1), *Loa loa* (XP_003139548.1), *Aedes aegypti* (XP_001655107.1), *Apis florea* (XP_003692456.1), *Canis familiaris* (XP_536291.3, XP_005618121.1), *Drosophila melanogaster* (NP 651365.1), *Dictyostelium discoideum* (XP_640350.1), *Dictyostelium fasciculatum* (XP_004351182.1), *Chaetomium thermophilum* (XP_006693771.1), *Monodelphis domestica* (XP_001364030.1), *Xenopus laevis* (NP 001086801.1, NP 001088220.1). Accession numbers for the various sequences in the NCBI Protein Database are given next to each species name. Numbers represent bootstrap values (after 1000 iterations) and the scale bar represents branch length as a fraction of the total tree length. The bootstrap values of >95% were displayed in the tree.

The full-length amino acid sequences of *Ss-*RIOK-2 and 14 other RIOK-2 homologues representing a range of different species were aligned and subjected to phylogenetic analyses (Fig. 2). There was concordance in topology between the MP, ML and NJ trees, which showed that *Ss-*RIOK-2 was grouped with RIOK-2s from other nematode species with absolute bootstrap support (100%) to the exclusion of molecules from organisms from other phyla. RIOK-2s from amphibians and mammals each formed separate clades, also supported by strong bootstrap values (99% and 100%, respectively, Fig. 2).

### Three-dimensional structural modelling of the *Ss-*RIOK-2 protein kinase

**Figure 3:**
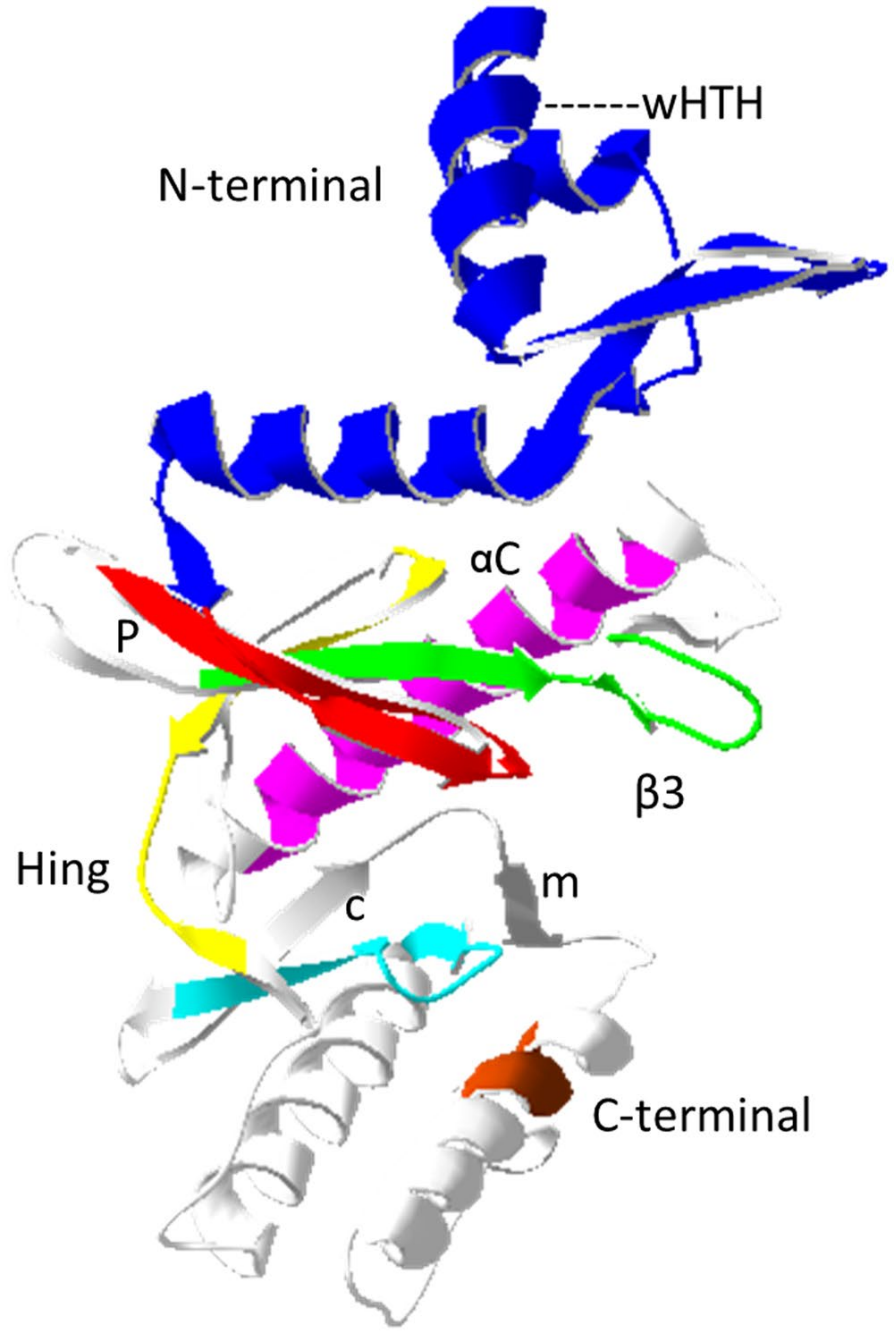
Homology model of the RIOK-2 protein kinase from *Strongyloides stercoralis*. The modeled structure of the *Ss-*RIOK-2 protein kinase shows different structural elements, N-terminal helix winged domain (blue), ATP binding motif (red), β3 (green), αC (pink), Hing (yellow), active site (cyan), metal-binding loop (grey) and αI (Amaranth).

To obtain the structural details and arrangement of these domains within *Ss-*RIOK-2 protein kinase, we modeled the structure of the *Ss-*RIOK-2 protein kinase using crystal structure of *C. thermophilum* RIOK-2 (*Ct-*RIOK-2) kinase as a template (PDB accession number 4GYG). Structural features present in *Ss-*RIOK-2 protein kinase are comparable to the structure of *Ct-*RIOK-2 with similar arrangements of structural elements (Fig. 3). The N-terminal wHWH domain is followed by an ATP binding site and a β-sheet as well as a long α helix, known as αC, followed by a long Hinge region and a C-terminal domain with catalytic and metal-binding loops, followed by a long α-helix (named αI), which is wedged between the N and C lobes close to the active site in eukaryotic *C. thermophilum*. Most of the residues were present within the allowed region of the Ramachandran plot (Supplementary Fig. S2B). ERRAT PLOT analysis of the final model shows that most of the residues are below 95% with a model structural quality factor of 90.612. Overall, no short contact was observed in the final model (Supplementary Fig. S2C). In a Verify 3D plot, 82.46% residues of the model are above 0.2 in the 3D/1D profile (Supplementary Fig. S2D).

**Figure 4:**
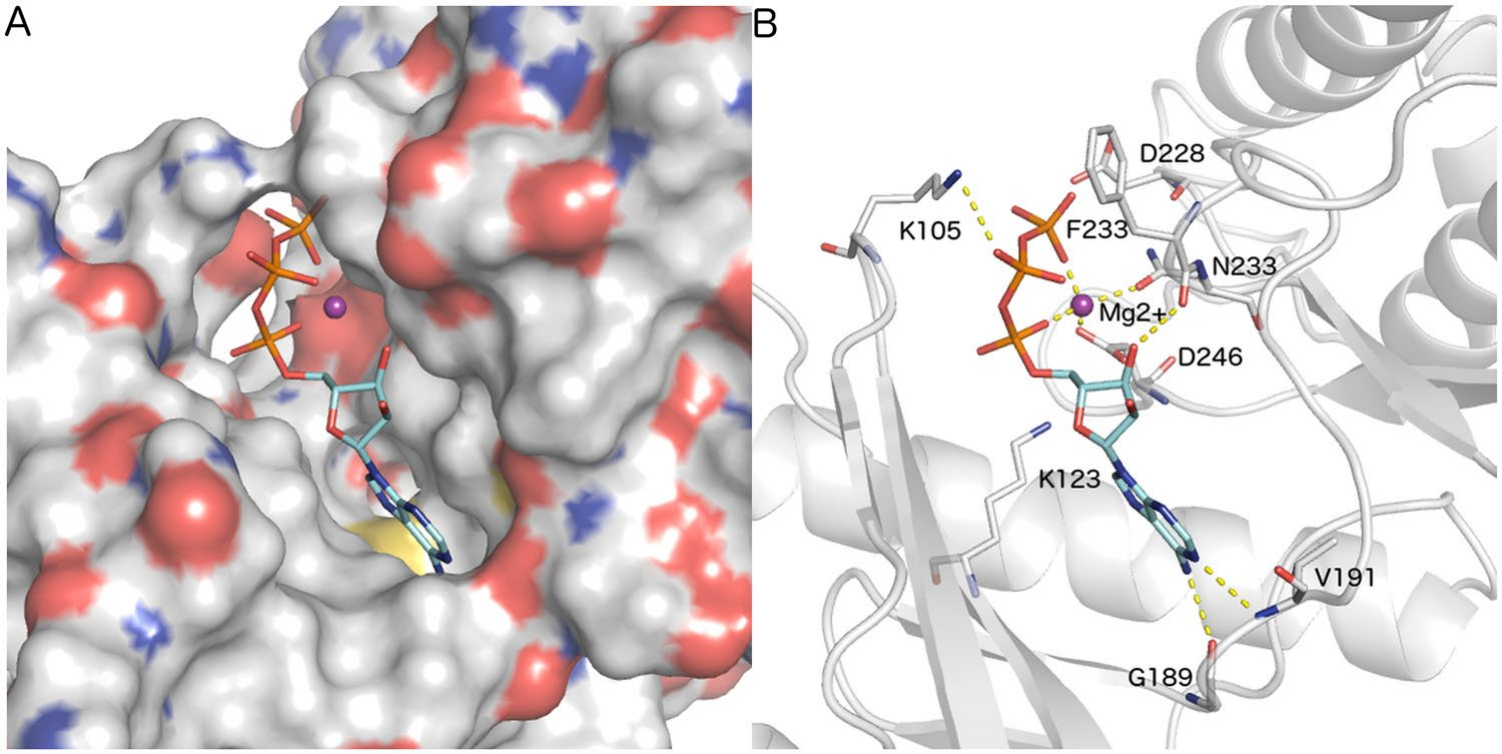
Surface structure of the *Ss*-RIOK-2 ATP binding pocket and interaction of ATP within modeled structure of *Ss-*RIOK-2-ATP. (**A**) Surface structure of the ATP binding pocket of *Ss-*RIOK-2-ATP. Blue is positive, red is negative, Mg^2+^ is shown as a purple sphere, ATP and catalytic residues are shown in stick representation. (**B**) Interaction of ATP with key residues present within the binding pocket of *Ss-*RIOK-2-ATP.

The docking simulation demonstrated that *Ss*-RIOK-2 has a conserved ATP binding site (Fig. 4A). Hydrophobic amino acid residues Met188, Gly189, Leu190, Val191 and Met235 form adenine subpocket within this site. The 6-NH_2_ of adenine is predicted to hydrogen bond (2.8 Å) to the main chain carboxyl group of Gly189. A weak hydrogen bond (3.2 Å) was also suggested between 1-N atom of adenine and main chain NH of Val191. The ribose moity of ATP was exposed to solvent and sandwiched by amino acid Ile109, Ile245 and Phe232. The 3′-hydroxyl of ribose oriented to the main chain carboxyl of Phe232 and hydrogen bonded (3.1 Å). Phosphate was in a highly polar and charged subpocket that included Lys105, Glu106, Lys123, Asn233, Asp246 and magnesium ions (Fig. 4B). The magnesium ion (purple sphere in Fig. 4B) was chelated by the negatively charged α and γ phosphates of ATP, Asn233 and Asp246 residues. The ionic interaction (3.2 Å) was predicted between the negatively charged β phosphate and positively charged Lys105 residue.

### Genomic organization of *S. stercoralis riok-2* and characterization of its putative promoter

**Figure 5:**
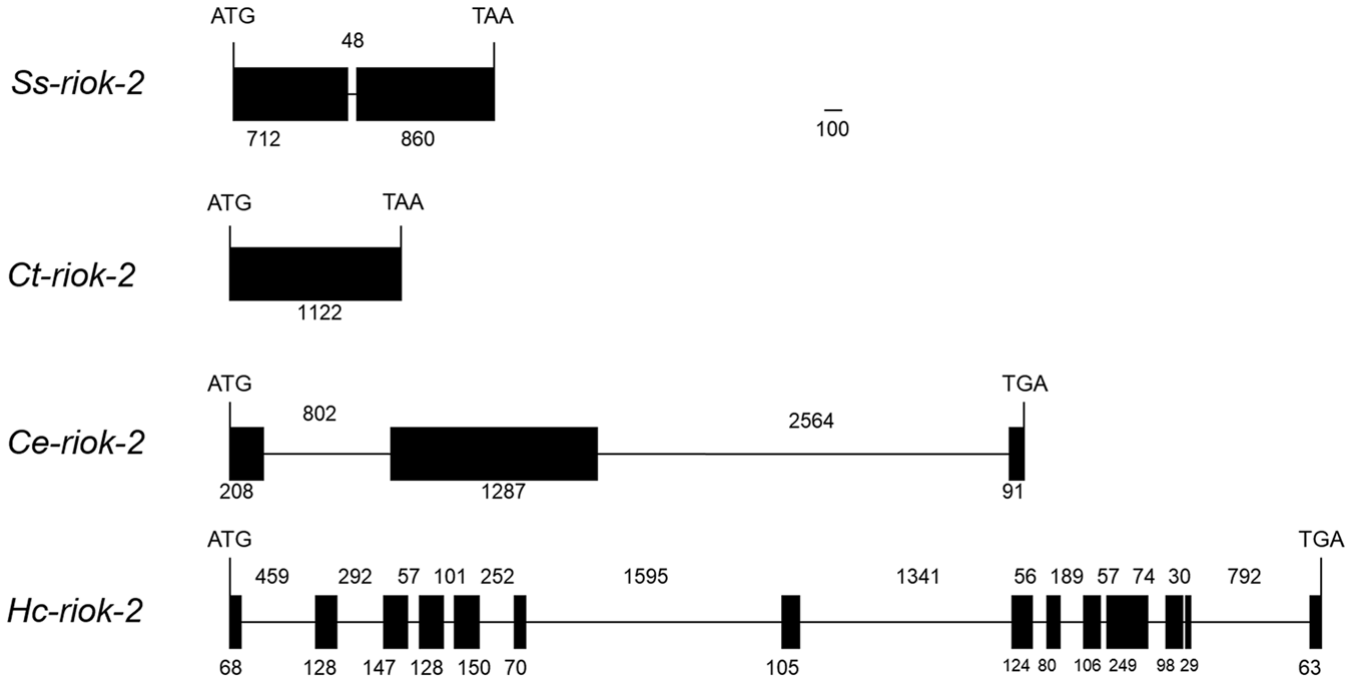
Diagrammatic representation of the genomic organizations of the riok-2 from *Strongyloides stercoralis* (*Ss-riok-2*) and orthologues from *Chaetomium thermophilum* (*Ct-riok-2*), *Caenorhabditis elegans* (*Ce-riok-2*) and *Haemonchus contortus* (*Hc-riok-2*). The organization of each gene was determined by aligning the cDNA and genomic DNA sequences, with intron-exon boundaries being defined using the GT-AG rule. Black boxes represent exons, whilst horizontal lines represent introns. Numbers below the boxes indicate the sizes of exons (in nucleotides), whereas numbers above the lines indicate the intron sizes.

The structures of the genes *riok-2* from *S. stercoralis* and its orthologues from *C*. *thermophilum*, *C. elegans* and *H. contortus* (Fig. 5) were inferred through separate alignments of genomic DNA sequences with cDNA sequences. These alignments revealed that *Ss-riok-2* is the shortest of the *riok-2* genes in these nematodes, comprising two exons and one intron and that, similar to *Ss-riok-2*, the *C. elegans* homologue (code Y105E8B.3) has one ORF (4953 nucleotides) comprising three exons and two introns. The spliced transcript of the *C. elegans* gene *riok-2* (1587 nucleotides) encoded a protein comprising 529 aa. By contrast, the alignment revealed that the *riok-2* of *H. contortus* (*Hc-riok-2*) is the longest and the most complex *riok-2* gene among the four species, comprising 6835 bp apportioned between 14 exons and 13 introns and encoding a transcript (code HQ198855.1) of 1587 nucleotides and a predicted protein 529 aa in length.

The predicted promoter of *Ss-riok-2* is 1308 bp in length, and the full length *Ce-riok-2* promoter sequence is 1269 bp in length. A sequence comparison revealed that the *Ss-riok-2* promoter is 51% identical to that of *Ce-riok-2* (Supplementary Fig. S3). The putative promoters of both genes are A + T rich, with the A + T content of 79.85% for *Ss-riok-2* and 62.49% for *Ce-riok-2*. A number of promoter elements, including eight TATA boxes, and one GATA (WGATAR) box, four inverse GATA (TTATC) boxes, three E-boxes (CANNTG), one CAAT (CCAAT) and one inverse CAAT (ATTGG) boxes are predicted within the *Ss-riok-2* promoter. In contrast, *Ce-riok-2* promoter region has one TATA box, one inverse GATA (TTATC) box, one E-box (CANNTG), two CAAT (CCAAT) and two GC boxes. Most of these motifs are dispersed across the promoters of the two genes, with no apparent pattern to their distribution.

### Transcriptional and expression profiles of *Ss-riok-2*

**Figure 6:**
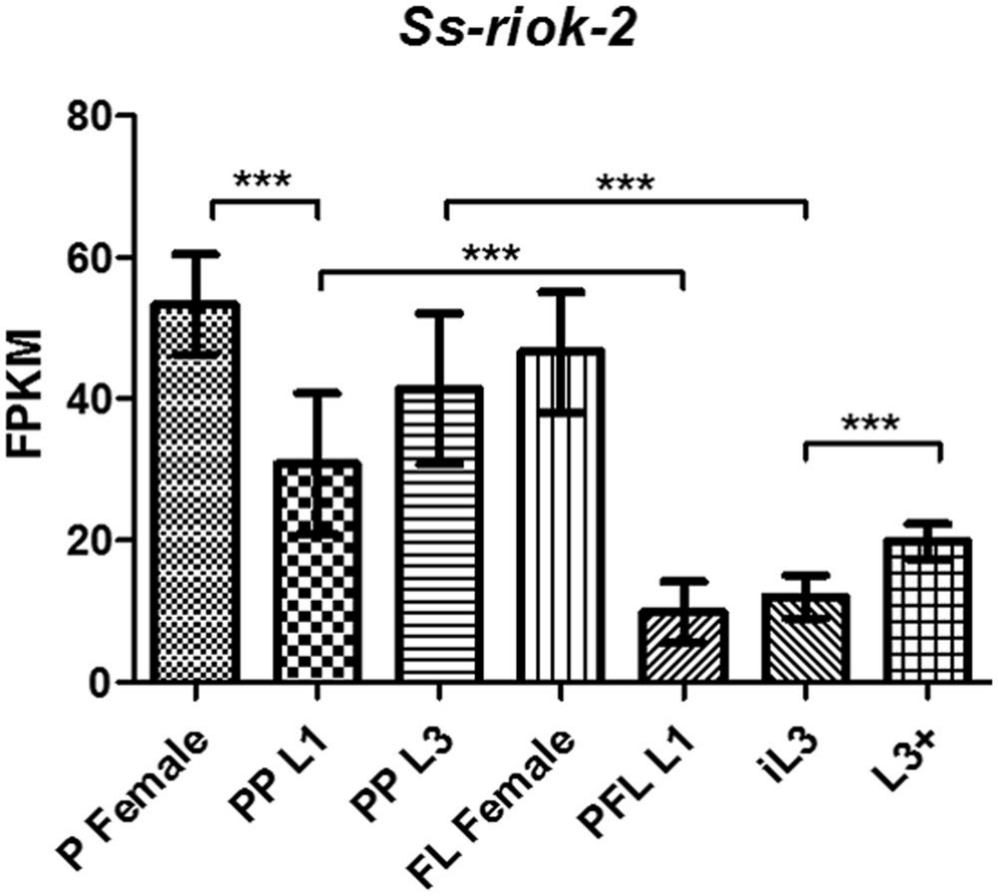
Transcriptional profile of *Ss-riok-2* in seven *Strongyloides stercoralis* life stages. Transcript abundances were compared in biological triplicate. Stages examined were: parasitic females (P Female), post-parasitic first-stage larvae (PP L1), post-parasitic third-stage larvae (PP L3), free-living females (FL Female), post-free-living first-stage larvae (PFL L1), infectious third-stage larvae (iL3), and *in vivo* activated third-stage larvae (L3+). Transcript abundances are expressed as fragments per kilobase of coding exon per million mapped reads (FPKM). Error bars represent 95% confidence intervals.

RNAseq revealed that *Ss-riok-2* is transcribed in all developmental stages of *S. stercoralis* with the highest transcript abundance in parasitic and free-living females and the lowest transcript abundance in post-free-living first-stage larvae (Fig. 6). Abundance of these transcripts increased significantly during the transitions from PFL L1 to iL3 and PP L1 to PP L3. In general, transcript abundance was significantly higher in post-parasitic stages, most of which develop heterogonically to free-living males and females in the UPD strain, compared with post-free living larval stages, which develop exclusively via the homogonic to iL3 in all *S. stercoralis*.

**Figure 7:**
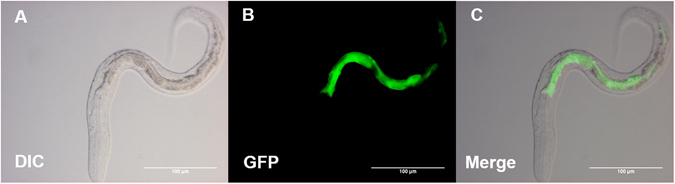
Representative expression pattern of GFP under the putative *Ss-riok-2* promoter in a transgenic second-stage larva of *Strongyloides stercoralis*. Differential interference contrast (DIC) and fluorescence (GFP) images showing the expression of construct *S. stercoralis Ss-riok-2p::gfp* (pRP2; Fig. S1) in a post-free-living second-stage larva. GFP reporter expression predominated in intestine. Scale bars = 10 μm.

To investigate the anatomical expression pattern of *Ss-riok-2*, we transformed parental free-living *S. stercoralis* females with construct pPR2 (Supplementary Fig. S1). Transformed females were paired with wild type *S. stercoralis* males and their post-free-living L1 progeny were screened for GFP fluorescence. Some of the immature eggs had GFP expression, even when they were still in the uteri of the free-living females (data not shown). GFP expression was most frequently observed in the intestine of transgenic L1 (Fig. 7) despite a varying intensity of GFP expression among different individuals.

### Autophosphorylation of *Ss-*RIOK-2

**Figure 8:**
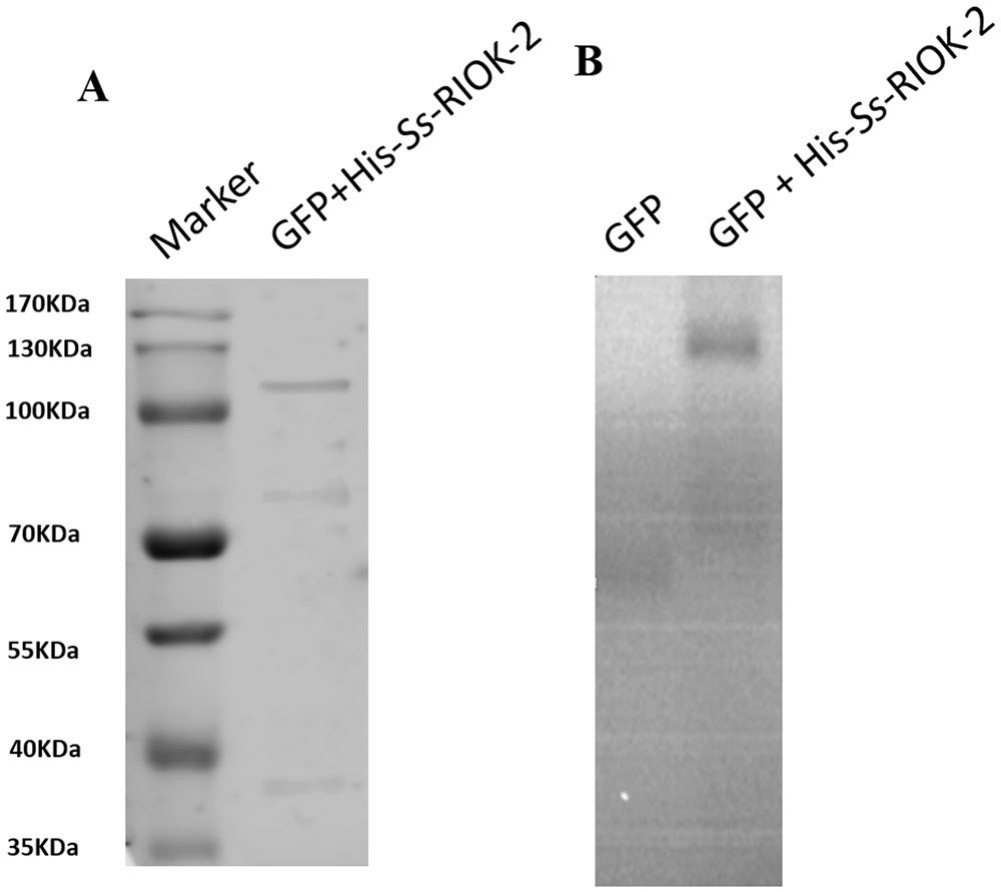
Autophosphorylation of recombinant His-*Ss-*RIOK-2. (**A**) Western-blot of His-*Ss-*RIOK-2 immunoprecipitated with His antibody. (**B**) Kinase assay showing autophosphorylation activities of recombinant His-*Ss-*RIOK-2.

As the natural substrate of *Ss*-RIOK-2 is unknown, we assessed its autophosphorylation activity. Recombinant His-tagged enzyme (designated His-*Ss*-RIOK-2) was expressed in *E. coli* (Fig. 8A). Purified recombinant His-*Ss*-RIOK-2 incubated with [γ^32^P] ATP produced radioactive signals, whereas purified recombinant His-GFP incubated with [γ^32^P] ATP didn’t produce radioactive signal, indicating that the His-*Ss*-RIOK-2 is capable of auto-phosphorylation (Fig. 8B).

## Discussion

Atypical serine/threonine RIOK-2 kinases are essential for viability of yeast, human cells and for *C. elegans* ^2, 9, 25–27^. In the present study, we determined the structure and discovered some functionalities of the *Ss-riok-2* gene in *S. stercoralis*, a parasitic nematode infecting human beings and dogs.

The catalytic cores of the majority of kinases are made up of 11 conserved subdomains similar to *Ct-*RIOK-2 (Fig. 3). The kinase domain in *Ss-*RIOK-2 is also shorter than the typical protein kinases present in eukaryotes. It lacks a critical activation loop (subdomain VIII), which was thought to be necessary for peptide recognition and binding^1^, and the last two helices of the C-terminal lobe (subdomain X and XI). The ATP binding pocket of *Ss-*RIOK-2 is well configured to dock with this molecule. The interaction between *Ss-*RIOK-2 and ATP features several strong interactions with atoms in the substrate (Fig. 4B). The conserved RIOK-2 signature sequence “GxGKES” in the ATP binding motif is slightly different from the signature sequences of RIOK-1 domains (with the sequence STGKEA) and RIOK-3 domains (with the sequence STGKES). The crystal structure of the fungal *Ct-*RIOK-2^15^ allows the study of the back pocket of the ATP-binding region and the design of specific compounds to bind these hydrophobic residues. In *P falciparum*, a number of protein kinase inhibitors were shown within the ATP binding domain in an in-silico docking simulation using *Pf-*RIOK-2 kinase as target enzyme^19, 20^. Therefore, this *Ss-*RIOK-2-ATP model could similarly be used to screen and identify some potent inhibitors by molecular docking. The 18 conserved residues of the flexible loop between β3 and αC of *Ss-*RIOK-2 are greatly disordered, making the sequence of this flexible loop distinct from those of the other RIO kinases. Thus, the function of this region may be different for each RIO kinase in an organism. Deletion of the flexible loop caused a slow-growth phenotype in yeast, particularly at lower temperatures^15^. Furthermore, the flexible loop is speculated to be a recognition or binding feature that orients the substrate and maintains its position during the catalytic phosphoryl transfer. *Ss-*RIOK-2 is capable of auto-phosphorylation (Fig. 8), a property of the RIOK-2 already described from *A. fulgidus*, *S. cerevisiae* and humans^2, 6, 7^. By analogy, *Ss*-RIOK-2 may also exclusively phosphorylate serines. The auto-phosphorylation site identified by phosphopeptide mapping and sequencing analysis was determined to be invariant at Ser 128 in *Af-*RIOK-2^28^. This residue is located on the flexible loop and within the sequence RLGRTSFRKIK in *Ss-*RIOK-2 and *Ce-*RIOK-2. The conserved catalytic regions in ePKs are thought to play key roles in transferring phosphate from ATP to substrate proteins. In *S. cerevisiae* RIOK-2, active site mutations indicate that RIOK-2 kinase activity is required for its dissociation from the late pre-40S particle and that it influences the progression and recycling of other late pre-40S processing factors. All these mutants supported cell growth but yielded a cold-sensitive phenotype in yeast^15^. The *Ss-*RIOK-2 catalytic loop was predicted by the model to interact between the conserved catalytic residue Asp-228 (Asp-229 in *S. cerevisiae*, is the proposed catalytic base for deprotonation of the substrate serine hydroxyl group in *S. cerevisiae* RIOK-2) and the metal-binding loop containing the conserved Asp-246 residue (Asp253 in *Sc-*RIOK-2 is responsible for binding to Mg^2+^), indicating that phosphoryl transfer from ATP to Asp 246 in *Ss*-RIOK-2 (Fig. 4B) is similar to that in *S. cerevisiae* RIOK-2.

Besides the active site, the conserved N- terminal domain (wHTH) of RIOK-2s also participates in some biological processes. In multiple sequence alignment, we identified a wHTH domain (1–98) in *Ss-*RIOK-2 (Fig. 1), the characteristic structure present in RIOK-2 kinase family. The wHTH domain is significant for its capacity as a DNA-binding protein, and also mediates protein-protein and protein-RNA interactions^10–12^. Such winged helices bind DNA through site-specific interactions of its major grooves with residues on -helix 3 of transcription factor HNF-3^29^ and specific interactions of W1, β2 and β3 with the major groove of the DNA in hRFX1^10^. Homologous conserved residues from the wHTH domain of *Ss-*RIOK-2 (Lys68, Arg91) suggest similar DNA binding properties.

In addition to the active sites and the N-terminal region, the C-terminal region of RIO domain consists of a kinase-inhibiting activity domain. This domain’s interaction with the ribosomal complex suggests this region as a target for the design of specific compounds that can inhibit RIOK activation or interactions. These findings suggest that *Ss-*RIOK-2 is an active protein kinase with functions that are similar to those of its homologues in yeast and humans, and it follows from this that *Ss-*RIOK-2 may represent a promising drug target.

*Ss-riok-2* has only one intron as compared to its homologues from *C. elegans* and *H. contortus* (Fig. 5)*. Ss-riok-2* gene is the shortest and has the fewest exons of the nematode *riok-2*s studied to date. Realizing that gene structure is not directly related to essential function, we nevertheless hypothesize that *Ss-riok-2* is required for transcriptional regulation by a complex mechanism. The 5′-UTR of *Ss-riok-2* is similar in length to that of *C. elegans riok-2*. The 5′-UTR of *Ss-riok-2* has 51% identity to that of the *Ce-riok-2* gene and also shares some promoter elements with the *C. elegans* homolog, though the elements’ similarity is limited (Fig. S3). Common elements in the promoters of *Ss-riok-2* and *Ce-riok-2* included TATA boxes, GATA and E-box. The TATA box is recognized by transcription factor IID and is necessary for regulation of transcriptional start point and the frequency with which the gene is transcribed^30, 31^. The GATA box is recognized by GATA transcription factors and is necessary for regulation of eukaryotic development and reproduction^32–36^. E-boxes are recognized and bound by basic helix–loop–helix (bHLH) proteins which regulate a wide range of developmental processes in eukaryotic organisms^37–41^. Conservation of these elements in *riok-2s* of *C. elegans* and *S. stercoralis*, as demonstrated here, suggests that their encoded kinases play an important role in gene transcription and expression in both free-living and parasitic nematodes.

To gain insight into the functions of *Ss-riok-2*, transcript abundance was measured in all developmental stages of *S. stercoralis*. *Ss-riok-2* transcripts occur throughout the life cycle (Fig. 6), suggesting that they function in the development of all stages of this parasite. In addition, the highest transcript abundance occurs in the parasitic and free-living females of *S. stercoralis*, suggesting that *Ss-riok-2* also plays important roles in the reproduction of this parasitic nematode. This is consistent with the transcriptional profiles of *riok-2* of *D*. *melanogaster* ^42^ and of two parasitic nematodes *B. malayi* ^43^ and *H. contortus* ^44, 45^ as well as of the free-living nematode *C. elegans*. Silencing of *Ce-riok-2* by RNAi leads to embryonic lethality and sterility, indicating that *riok-2* is involved in nematode embryonic development and reproduction. Interestingly, after iL3 activation, abundance of *Ss-riok-2* transcripts is significantly higher in L3 + than in iL3, suggesting that RIOK-2 activity is necessary for *S. stercoralis* larvae to resume development in the host. Furthermore, an obvious trend seen in *S. stercoralis* transcript abundance profiles is that they are significantly higher in heterogonically developing larvae (those going to free-living adults) than in homogonically developing larvae (those going to L3i), suggesting that *Ss-riok-2* may have an important function in the reproductive development of *S. stercoralis*’s free-living adult stages, again supporting its role in the reproduction of this parasite.

Preliminary functional studies of *riok-2* genes in non-nematode species have revealed roles in ribosome maturation and cell cycle progression, indicating that *riok-2* genes are required for viability and fertility. In order to explore the function of *Ss-riok-2* in various tissues in *S. stercoralis*, the anatomical expression pattern was analyzed by expressing a transcriptional reporter based on *Ss-riok-2* in post free-living larvae of this parasite. Strong intestinal expression under the *Ss-riok-2* promoter was observed (Fig. 7). Intestinal expression is controlled by the ELT-2 transcription factor in *C. elegans* ^46, 47^. A search of the 1.5 kb region upstream of the *Ss-riok-2* translational start site for ELT-2 recognition motifs revealed five characteristic TGATAA motifs, indicating that intestinal expression of *Ss-riok-2* may depend on an ELT-2-like transcription factor. This expression profile might not only identify spatio-temporal functionalities of the promoter, but also hint at specific functions of *Ss-*RIOK-2. In *C. elegans*, the intestine is a large organ that carries out diverse functions executed by multiple distinct organs in higher eukaryotes, including digestion of food, absorption of processed nutrients, synthesis and storage of macromolecules, initiation of an innate immune response to pathogens, and nurturing of germ cells by producing yolk^48–51^. Nematode intestinal cells are very large and contain large nuclei with prominent nucleoli, many mitochondria and extensive rough endoplasmic reticulum with many ribosomes. RIOK-2 enzymatic activity is crucial for cleavage of 20 S pre-rRNA and ribosome biogenesis^1, 2^. In this aspect, expression of *Ss-riok-2* in the intestine suggests that *Ss-*RIOK-2 also has an important function in ribosome biogenesis for *S. stercoralis* and underscores the potential of this protein as a therapeutic target. In addition, the digestive and metabolic activities of the intestine are central to the growth and development of any organism. The *C. elegans* intestine is an important endocrine tissue^52^. The yolk proteins are synthesized by and secreted from the intestine into their destination in the gonad^48, 51, 53^. A large-scale double-stranded RNA interference (RNAi) study of *C. elegans* showed that the silencing of *Ce-riok-2* leads to sterility and embryonic lethality. Therefore, *Ss-*RIOK-2 may play an important role in development of eggs by interacting with yolk proteins in *S. stercoralis*.

In summary, this study isolated and characterised the cDNA, genomic DNA and promoter of *Ss-riok-2*, which encode the RIOK2 protein kinase in the parasitic nematode *S. stercoralis*. *Ss-*RIOK-2 contains a RIOK-2 signature motif (wHTH) and has high similarity with a range of homologues from different species. The predicted formation of a stable *Ss-*RIOK-2-ATP complex supports the feasibility of exploiting this novel allosteric site of *Ss-*RIOK-2 as a potential anti-parasite drug target. Recombinant *Ss-*RIOK-2 has kinase activity. We also ascertained its transcriptional profile in seven key developmental stages, finding the highest transcript abundance in parasitic and free-living females and confirming its localization in the intestine of this parasite. The characterization and temporal and spatial expression patterns of the RIOK-2 in *S. stercoralis* lay the foundation for exploring the function of RIOK-2 in the biological processes of parasitic nematodes.

## Materials and Methods

### Ethics statement

No human subjects were used in these studies. The *S. stercoralis* (UPD strain) was maintained in prednisolone-treated Beagles in accordance with a protocol (Permit Number: SYXK-0029) approved by the Committee on the Ethics of Animal Experiments of Hubei Province. The care and maintenance of animals were in strict accordance with the recommendations in the Guide for the Regulation for the Administration of Affairs Concerning Experimental Animals of the P. R. China.

### *S. stercoralis* strains, and their maintenance

The *S. stercoralis* UPD strain was maintained in immuno-suppressed dogs and cultured as previously described^54^. UPD strain iL3 for nucleic acid extraction and experimental infections in dogs were isolated via the Baermann funnel technique from seven-day-old charcoal coprocultures. The iL3s were washed several times with a sterile buffered saline called BU buffer^54^. Free-living females for microinjection were transferred from the Baermann apparatus (along with a minimal fluid volume) to Nematode Growth Medium (NGM agar) plates seeded with *Escherichia coli* OP50^54^. All cultures of *S. stercoralis* were incubated at 22 °C unless otherwise noted.

### Genomic DNA and RNA preparation

*S. stercoralis* genomic DNA was extracted from 10,000–20,000 iL3s by sodium proteinase K treatment^55^, followed by purification using an EasyPure Genomic DNA Kit (TransGen Biotech, Beijing, China). DNA samples were stored at −20 °C until use. RNA was extracted from 20,000~30,000 iL3s using the Trizol reagent (Life Technologies, USA) according to the manufacturer’s protocol. RNA integrity and yields were verified by electrophoresis and spectrophotometry (Nano Drop Technologies, Thermo Scientific, USA), respectively, and 1 μg of RNA was reverse transcribed to cDNA using a kit (Smart RACE Kit, Clontech, USA). Extracted total RNA was treated with RQ1-RNase-Free DNase (Promega, USA) and then stored at −80 °C until use.

### Isolation of the full-length *Ss-riok-2* cDNA

The genomic sequence of *Ss-riok-2* was determined using two highly degenerate primers *riok-2-Bam*HI/F and *riok-2-Sal*I/R (S1 Table) with restriction sites to amplify a coding sequence with homologies to *H. contortus* (GenBank: HQ207527) and to *C. elegans* (GenBank: NM_061143) from cDNA synthesised from total RNA extracted from iL3s. PCR (50 μl) was performed in 10 mM Tris–HCl, pH 8.3, 50 mM KCl, 4 mM MgCl_2_, 250 μM each of dNTP, 100 pmol of each primer and 1 U Taq polymerase (TaKaRa, Japan) under the following conditions: initial denaturation at 94 °C for 5 min; then 30 cycles of denaturation at 94 °C for 30 s, annealing at 57 °C for 30 s and extension at 72 °C for 2 min, with a final extension at 72 °C for 10 min. This PCR product was electrophoresed and visualised on a 1% agarose gel from which a single, major band with little background was excised, cloned into the pMD19-T vector (Takara Biotechnology) and sequenced.

### Isolation of the putative promoter of *Ss-riok-2*

Template genomic DNA extracted from *S. stercoralis* iL3s, and a pair of primers with restriction sites (*Ss-riok-2-Pst*I/F and *Ss-riok-2-Sma*I/R, S1 Table) were designed to amplify the *Ss-riok-2* promoter sequence using the following PCR cycling conditions: initial 94 °C, 5 min; then 94 °C, 30 s, 65 °C, 2 min for 35 cycles; final extension at 65 °C for 10 min. The PCR product was electrophoresed and visualised on a 1% agarose gel from which a single, major band with little background was excised, cloned into pMD19-T vector (Takara Biotechnology) and sequenced.

### Bioinformatic and phylogenetic analyses

The sequence of *Ss-riok-2* was compared by BLASTx^37^ with sequences in non-redundant databases from NCBI (http://www.ncbi.nlm.nih.gov/) to confirm the identity of genes isolated. Conceptual translation of *Ss-riok-2* cDNA into predicted amino acid (aa) sequences was performed using the free software package Bioedit (http://www.mbio.ncsu.edu/BioEdit/bioedit.html#downloads). The protein motifs of *Ss-*RIOK-2 were identified by scanning the databases PROSITE^56^ (www.expasy.ch/tools/scnpsit1.html) and Pfam^57^ (www.sanger.ac.uk/Software/Pfam/). The aa sequence alignments were carried out with the homologues from selected species using the program MAFFT 7.0^58^ (http://mafft.cbrc.jp/alignment/software/), and adjusted by manual inspection. The functional domains and subdomains of *Ss-*RIOK-2 were identified in the protein alignment. Promoter elements in the 5′-UTR were predicted using the transcription element search system Matrixcatch (http://www.gene-regulation.com/cgi-bin/mcatch/MatrixCatch.pl)^59^.

The aa sequences inferred from *Ss-riok*-*2* and 14 selected sequences from homologues in other invertebrates and vertebrates were subjected to phylogenetic analysis by Clustal X^60^ and manually adjusted. The species selected were five nematodes, including *Ascaris suum* (ADY41687.1), *Caenorhabditis elegans* (NP_493544.2), *Caenorhabditis remanei* (XP_003097720.1), *Chaetomium thermophilum* (XP_006693771.1), *Haemonchus contortus* (ADW23593.1), *Loa loa* (XP_003139548.1), and eight non-nematode species, including *Aedes aegypti* (XP_001655107.1), *Apis florea* (XP_003692456.1), *Canis familiaris* (XP_536291.3, XP_005618121.1), *Drosophila melanogaster* (NP 651365.1), *Dictyostelium discoideum* (XP_640350.1), *Dictyostelium fasciculatum* (XP_004351182.1), *Monodelphis domestica* (XP_001364030.1), *Xenopus laevis* (NP 001086801.1, NP 001088220.1). The phylogenetic analysis was conducted using the neighbour-joining (NJ), maximum parsimony (MP) and maximum likelihood (ML) methods based on the Jones-Taylor-Thornton (JTT) model^61^. Confidence limits were assessed by bootstrapping using 1,000 pseudo-replicates for NJ, MP and ML, and other settings were obtained using the default values in MEGA v.5.0^61^. A 50% cut-off value was implemented for the consensus tree.

### Molecular modeling

The three-dimensional structure of the RIOK-2 kinase from *Chaetomium thermophilum* (*Ct-*RIOK-2 kinase; PDB code 4GYG) was used to create a homology model of *Ss-*RIOK-2 with the program DeepView Swiss-PdbViewer and the SWISS-MODEL server^62^. The crystal structure of RIOK-2 (native form) from *C. thermophilum* was suitable with 52.19% identity in the ~155 residues (25–321) in *S. stercoralis* (see ref. 15). Remaining residues of *Ss-*RIOK-2 (approximately 368 residues) did not show significant similarity and did not contain the sites of ATP binding motif. Hence, this part was not taken for homology modeling. All models were energy minimized by Swiss PDB VIEWER 4.1.0, and structural quality of the refined models was again assessed by PROCHECK^62^. The structural quality of final model was verified by Verify 3D, ERRAT plot, Procheck, and Ramachandran plot^63, 64^.

Given the importance of magnesium in RIOK-2 catalysis, one magnesium ion was artificially introduced into the empirical phosphate binding site of the model by comparison with the *C. thermophilum Ct-*RIOK-2 structure co-crystalized with ADP (PDB code: 4GYI). Meanwhile an ADP was “borrowed” from *C. thermophilum* kinase *Ct-*RIOK-2 and deployed in the homology model after structure superimposition.

Binding of ATP to the *Ss-*RIOK-2 protein kinase active site was evaluated using the docking suite Glide_XP^65^. This software elaborates low-energy poses when searching the conformational space of the ligand while the protein remained rigid based on enthalpy interactions. The possible ionic species states of ATP at pHs ranging from 5.0 to 9.0 were generated by LigPre in Maestro 2015. The Schrödinger suite protein preparation wizard was used to assign atom type and side chain protonation states^66^. A modelled *Ss-*RIOK-2 protein structure was prepared using the Protein Preparation Wizard panel^66^ prior to use. Docking simulations were run using default parameters, and Glide receptor grids were generated by defining a 10 Å box localized at the centroid of ADP.

### Transcript abundance based on RNA-seq analysis

The *S. stercoralis* PV001 line, derived from a single female worm of the UPD strain was used to derive transcriptomic data for the following stages of the parasite: parasitic females (P Female), post-parasitic first-stage larvae (PP L1), post-parasitic third-stage larvae (PP L3), free-living females (FL Female), post free-living first-stage larvae (PFL L1), infective third-stage larvae (iL3), *in vivo* activated third-stage larvae (L3+). These data are publicly available^67, 68^. Transcript abundances were quantified using RNAseq^67^ and calculated using Cufflinks v.2.0.2 (http://cufflinks.cbcb.umd.edu/) as fragments per kilobase of coding exon per million fragments mapped (FPKM), with paired-end reads counted as single sampling events^69^. FPKM values for coding sequences (CDS), ± 95% confidence intervals, were calculated for each gene using Cuffdiff v.2.0.2^70, 71^. *P*-values ≤ 0.05 were considered statistically significant, and *P*-values ≤ 0.001 were considered statistically highly significant. FPKM and 95% confidence intervals were plotted in Prism version 5.01 (GraphPad Software, Inc., http://www.graphpad.com/).

### Protein expression and purification

A full-length cDNA of *Ss-riok-2* was PCR amplified using primers *Ss-riok-2*-*Nde*I and *Ss-riok-2-Xho*I (S1 Table). The PCR product was then cloned into pMD19-T and sequenced, and further subcloned into the vector pET-42b-*gfp*. The insert of the recombinant plasmid pET-42b*-gfp-riok-2* was sequenced and its open-reading frame (ORF) encoding the fusion protein GFP-His-*Ss*-RIOK-2 was confirmed. This construct was used to transform *E. coli* (*Transetta*; Transgene) cells for protein expression. The bacterial cells were diluted 1:100 into new LB/Kna^+^ medium, after 3 h of growth at 37 °C. The expression cultures were induced with IPTG (1:1000), grown at 28 °C and 150 rpm/min agitation overnight and then harvested by centrifugation at 10000 rpm for 2 min. The bacteria were re-suspended in 50 mM Tris-Cl with 0.1 M NaCl, passed through a 0.45 μm filter and loaded onto 1 mL His Trap FF affinity columns (GE Healthcare). The bound *Ss*-RIOK-2 was eluted with 50 mM Tris-HCl and 40 mM reduced glutathione, pH 8.0. The eluate was concentrated using Ultra-15 50 KD centrifugal filter devices (Millipore). The final concentration was 1 mg/mL.

### Kinase assays

The assay for autophosphorylation was carried out in a reaction buffer containing 25 mM Tris pH 7.5, 50 mM NaCl, 2 mM MgCl_2_ and 1 μCi [γ^32^P] ATP^72^. 10 μg purified GFP-His-*Ss*-RIOK-2 were added into the autophosphorylation reaction. Control reactions consisted of all assay components but replacing GFP-His-*Ss-*RIOK-2 with GFP. The reaction mixtures were incubated at 37 °C for 1 h 30 min. Reactions were terminated by the addition of gel loading buffer and run on SDS polyacrylamide gel for 1 h at 120 volts. Radiolabeled proteins in the gel were detected by autoradiography.

### Preparation of transgene constructs

Plasmid vectors for transformation of *S*. *stercoralis* were prepared by first amplifying the *Ss-riok-2* promoter sequence from *S*. *stercoralis* genomic DNA with forward and reverse primers including restriction site sequences for *Pst*I and *Sma*I, respectively (Thermo). The amplicon was gel-purified using the Tiangen Gel purification kit (Tiangen Biotech). The purified product was then subcloned into the promoter-less vector pAJ01 upstream of the *gfp* coding sequence^73^ (Addgene, Cambridge, Massachusetts, USA, http://www.addgene.org) to create the plasmid pRP2 (Fig. S1). The plasmid was sequenced and transformed into *E*. *coli* (DH5α; Transgene) and amplified in the bacteria cells at 37 °C. After 8 h of growth, the construct was extracted by TIAN pure Midi Plasmid Kit (Tiangen Biotech) and diluted to 25 ng/μL and 50 ng/μL. The diluted construct was stored at −20 °C.

### DNA transformation

Standard methods for transforming *S. stercoralis* by microinjecting plasmid constructs into gonads of free-living females were carried out as described^74^. In brief, free-living *S. stercoralis* females were immobilized on dry agar pads overlain with halocarbon oil, and a solution of 25 ng/μL of plasmid *Ss-riok-2p::gfp::Ss-era-1t* (pRP2) in injection buffer was injected into the gonad between the distal tip cell and the bend using finely drawn glass micropipets pressurized with air at 20 psi. Microinjected female worms were transferred singly, along with one or two free-living males, to NGM agar plates with lawns of *E. coli* OP50, and plates were sealed with parafilm and incubated at 22 °C. Microinjected *S. stercoralis* and their progeny were observed daily. Initial screening for *gfp* expression in F1 progeny was done at intervals of 24 h and 48 h following injection using a stereomicroscope outfitted with a coaxial epifluorescence unit with a GFP LP filter. Larvae expressing GFP were anesthetized using 20–50 mM levamisole (Sigma-Aldrich), and transferred to 2% agar pads (Sigma-Aldrich) on standard microscope slides^68^. Worms with GFP expression were examined in detail using a compound epifluorescence microscope equipped with Differential Interference Contrast (DIC) optics and a digital camera (Olympus BX51, Japan).

## Electronic supplementary material

Supplementary Information
