# Supplementary material for: Structural and developmental expression of Ss-riok-2, an RIO protein kinase encoding gene of Strongyloides stercoralis
Source: Sci Rep. 2017 Aug 18;7:8693. doi: 10.1038/s41598-017-07991-2 (PMC5562798; doi:10.1038/s41598-017-07991-2)
Supplement: Supplementary file 1 — Supplementary Information [file 41598_2017_7991_MOESM1_ESM.pdf]

**Supplementary Information**

# Structural and developmental expression of *Ss-riok-2*, an RIO protein kinase encoding gene of *Strongyloides stercoralis*

Wei-Qiang Lei[1], James B. Lok[2], Wang Yuan[1], Yue-Zhou Zhang[1], Jonathan D. Stoltzfus[2,3], Robin B. Gasser[4], Si-Yuan He[1], Huan Zhou[1], Rui Zhou[1], Jun-Long Zhao[1], Min Hu[1]*

**Supplementary Figures:**

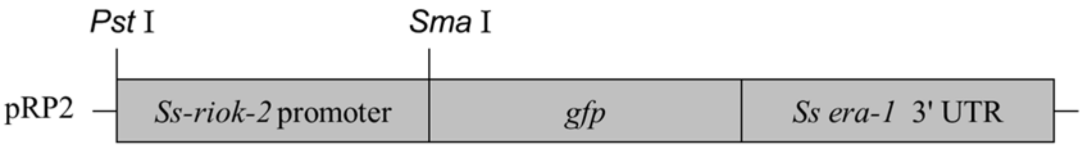


**Supplementary Fig. S1: Cloning strategy for reporter construct.** The constructs containing the 1280 bp promoter of *Ss-riok-2* (pRP2) were made based on PAJ01. The restriction sites for *Pst*I and *Sma*I were marked on the diagram, respectively. UTR, untranslated region.

A

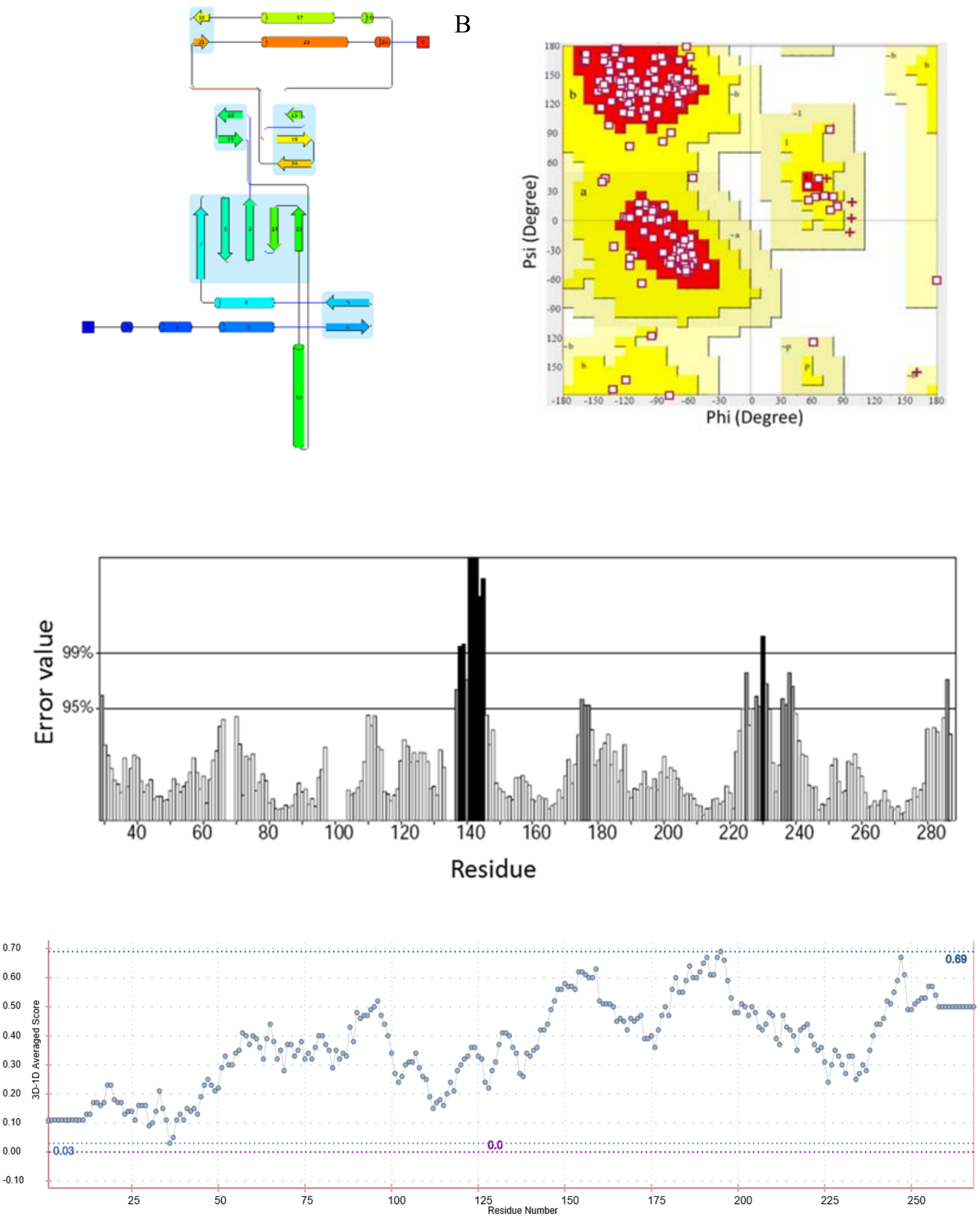

**Supplementary Fig. S2: Quality of modeled *Ss*-RIOK-2 protein kinase structure.** (A) Topology diagram of *Ss*-RIOK-2 protein kinase to depict the organization of structure elements. The *Ss*-RIOK-2 protein kinase model was validated by (B) Ramachandran plot (C) Errat plot and (D) Verify 3D. Analysis indicates that modeled structure was of high quality.

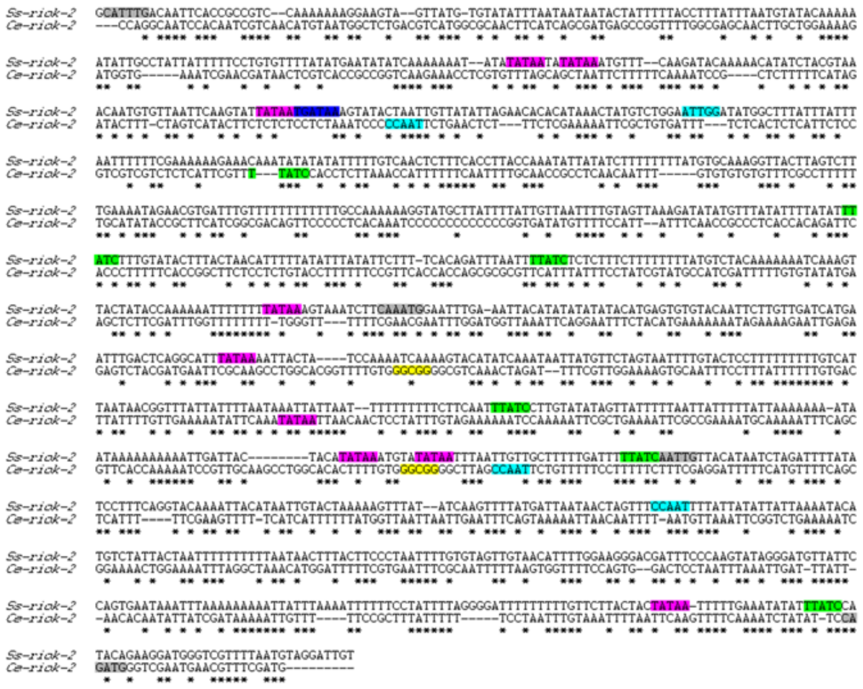

**Supplementary Fig. S3: Alignment of promoter regions predicted from the sequences of the 5'-UTRs of *Ss-riok-2* and *Ce-riok-2*.** Highlighted in different colours are predicted promoter elements: CAAT (CCAAT) or inverse CAAT (ATTGG) motif (turquoise); GATA (WGATAR) (blue); inverse GATA (TTATC) (green); GC box (yellow); E-box (CANNTG) (grey); TATA box (pink).

**Supplementary Table S1: PCR Primers used to amplify coding sequence and promoters for synthesis of transformation constructs**

| Element | Primer designation | Sequence |
|---|---|---|
| *Ss-riok-2* coding sequence | *Ss-riok-2-Bam*HI/F | 5'-GC<u>GGATCC</u>ATGGGTCGTTTTAATGTAGGATTG-3'[a] |
| | *Ss-riok-2-Sal*I/R | 5'-GC<u>GTCGAC</u>TTAAAACCATCCTGAATATTCT-3'[b] |
| *Ss-riok-2* coding sequence | *Ss-riok-2-Nde*I/F | 5'-<u>CATATG</u>ATGGGTCGTTTTAATGTAGGATTGT-3'[c] |

| | | |
|---|---|---|
| | *Ss-riok-2-Xho*I/R | 5'-CTCGAGGTAAAACCATCCTGAATATTCTTTT-3'[d] |
| *Ss-riok-2* promoter | *Ss-riok-2-Pst*I/F | 5'-CAACTGCAGGCATTTGACAATTCACCGCCG-3'[e] |
| | *Ss-riok-2-Sma*I/R | 5'-TCCCCCGGGACAATCCTACATTAAAACGACCCAT-3'[f] |

[a] *Bam*HI restriction site underscored

[b] *Sal*I restriction site underscored

[c] *Nde*I restriction site underscored

[d] *Xho*I restriction site underscored

[e] *Pst*I restriction site underscored

[f] *Sma*I restriction site underscored
